# Supplementary material for: A nonlinear relationship between prediction errors and learning rates in human reinforcement-learning
Source: PLoS Comput Biol. 2025 Sep 12;21(9):e1013445. doi: 10.1371/journal.pcbi.1013445 (PMC12449023; doi:10.1371/journal.pcbi.1013445)
Supplement: S1 Text — Fig A. The relationship between environmental noise and parameter κ. Fig B. The average magnitude of prediction errors under different learning models. Fig C. A nonlinear relationship between the absolute magnitude of prediction errors and learning rates. Fig D. Learning rate acceleration differences between hybrid Pearce-Hall, cubic and exponential-logarithmic models. Fig E. Prediction errors encode the structure of the environment. Fig F. Behavioural results from the in-lab experiment. Fig G. The results of simulations highlighting the descriptive features of environments which favour one model over the others. Fig H. Supplementary control analysis of the pupil dilation with the outputs of the Pearce-Hall model. Fig I. Outputs of the generate-recover simulations. (PDF) [file pcbi.1013445.s001.pdf]

Supplementary Tables and Figures

Table A: Demographic details of participants

Measure	Mean (SD)
**Age**	29.4 (9.79)
**Gender**	56.25% Female
**Years of Education**	17.72 (2.00)
**State-STAI**	30.13 (7.36)
**Trait-STAI**	35.88 (7.61)
**QIDS-16**	6.22 (4.33)

QIDS-16; Quick Inventory of Depressive Symptoms, 16 item self-report version. Trait/State-STAI; Spielberger State Trait Anxiety Inventory. Note that scores of 6 or above on the QIDS-16 indicate the presence of mild depressive symptoms. The Trait/State-STAI has no standard cut-off scores.

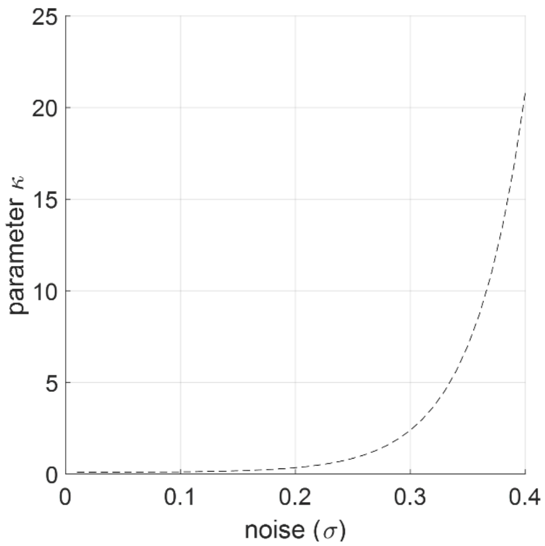

Supplementary Figure A. The relationship between environmental noise and parameter κ which underlies Figure2.

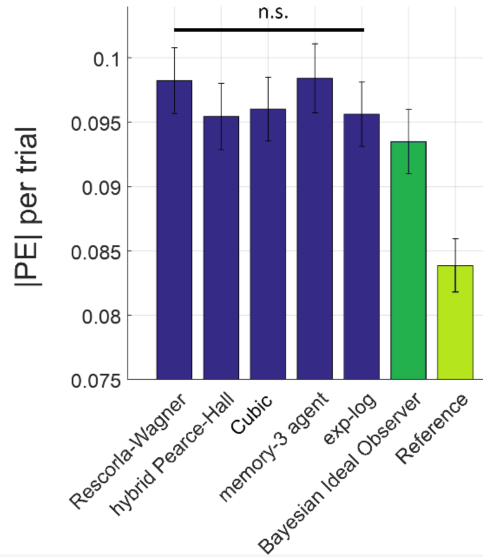

Supplementary Figure B. The average magnitude of prediction errors (|PE| on y-axis) under different learning models. Across 1500 trials in the simulated task environment (Figure 4), the RL models lead to average PEs which were not statistically significantly different. Based on an exponential relationship between environmental noise and the free-parameter κ (Figure 2), any memory-n agent (i.e. computing the expected uncertainty from the standard deviation of outcomes observed in the environment based on a history of n previous trials) can adjust its learning behaviour dynamically. An example is shown for a memory-3 agent who computes the standard deviation of outcomes based on the observations in the past 3 trials. The behaviour of a recently described 5-dimensional Bayesian Ideal Observer model[1-3] is shown for illustrative purposes (dark green bar). The “Reference” bar (light green) refers to |PE|s computed with respect to the mean of the generative process (i.e. if an agent had all the information about the environment up front).

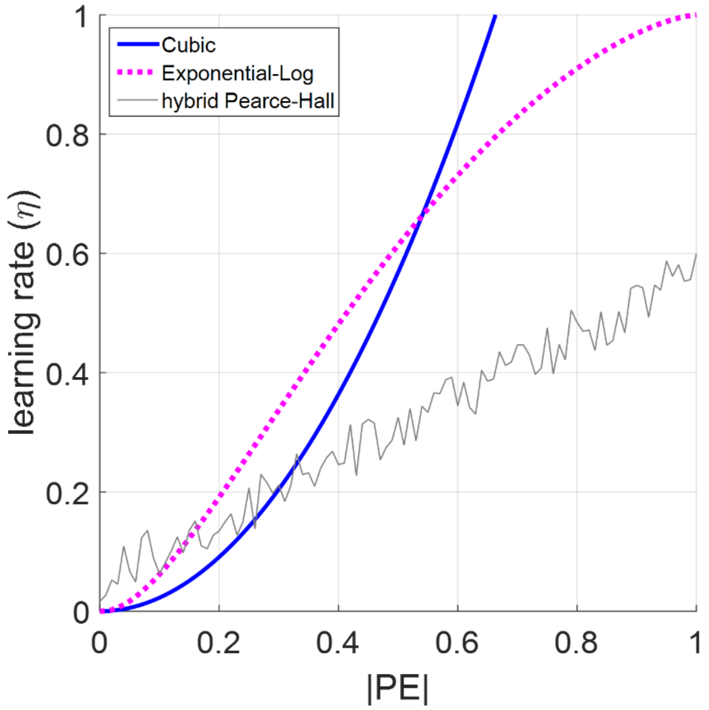

Supplementary Figure C. A nonlinear relationship between the absolute magnitude of prediction errors (|PE|) and learning rates. The exponential-logarithmic model which behaves marginally, but not statistically, better in the simulation environment (Figure 4) have steeper learning rates relative to the cubic model for observations leading to PEs ≤ 0.50. The maximum PE magnitude (i.e. the largest difference between any n-(n-1) outcomes) was 0.71 in the simulation environment. Therefore, the trajectory of the curves above |PE|>0.71 on x-axis is theoretical. The hybrid Pearce-Hall model would suggest a stochastic linear relationship between |PE|and learning rates, as the learning rate on a given trial is also a function of the learning rate from one previous trial. The value of the ω parameter, that is 0.48, suggests that the learning rates would be influenced by the magnitude of the prediction errors to a greater extent leading to the observed linear relationship (Eq.2).

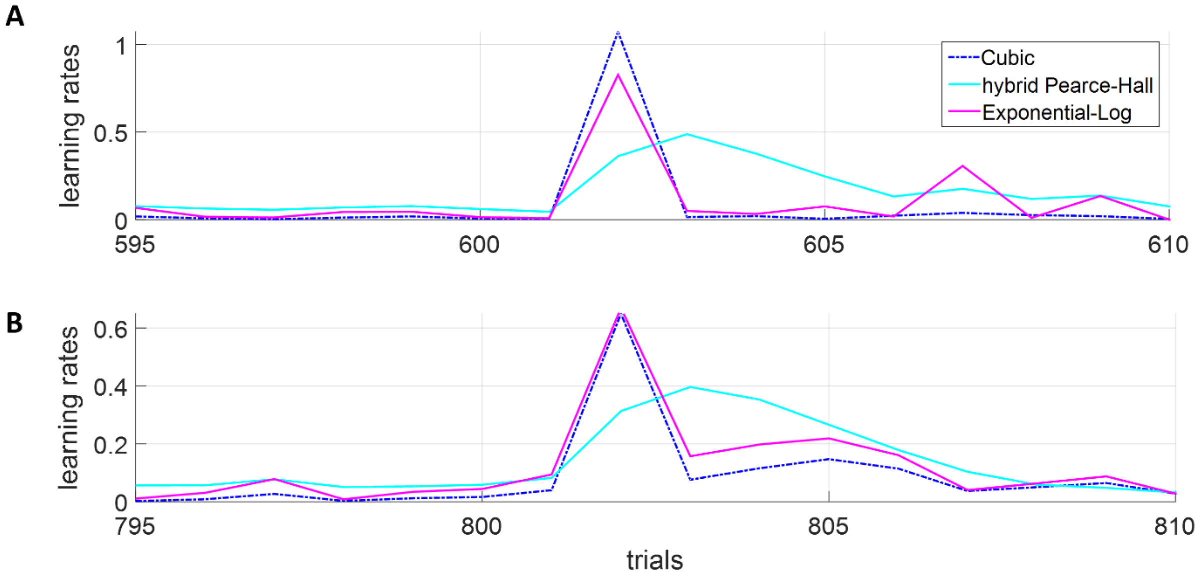

Supplementary Figure D. Learning rate acceleration differences between hybrid Pearce-Hall, cubic and exponential-logarithmic models. The hybrid Pearce-Hall model is slower to accelerate and decelerate learning rates. The trial numbers on x-axes refer to the task environment shown in Figure 4. Examples are shown for **(A)** transitions within high volatility high noise, and **(B)** between high volatility high noise and low volatility low noise periods**.** In tasks designs with explicit presentation of outcomes (e.g. Figure 4 in which exact outcome magnitudes are shown in the task environment) learning rates can marginally be higher than 1[4,5].

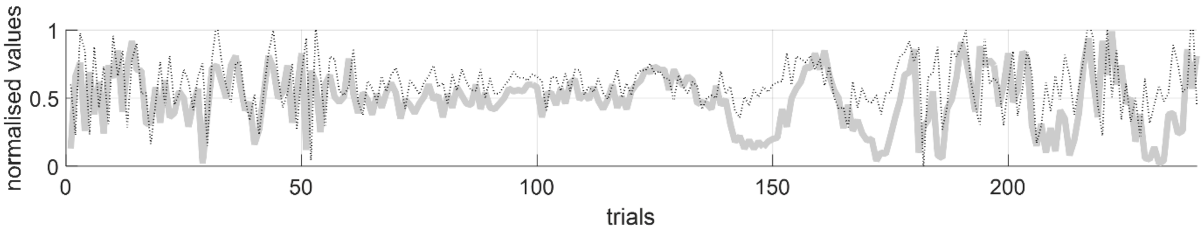

Supplementary Figure E. Task environment of the reward magnitude learning task (thick grey line) as experienced by a participant. Block orders were randomised. Prediction errors from the exponential-logarithmic model superimposed (thin dashed black line), demonstrating that prediction errors globally encode the structure of the environment.

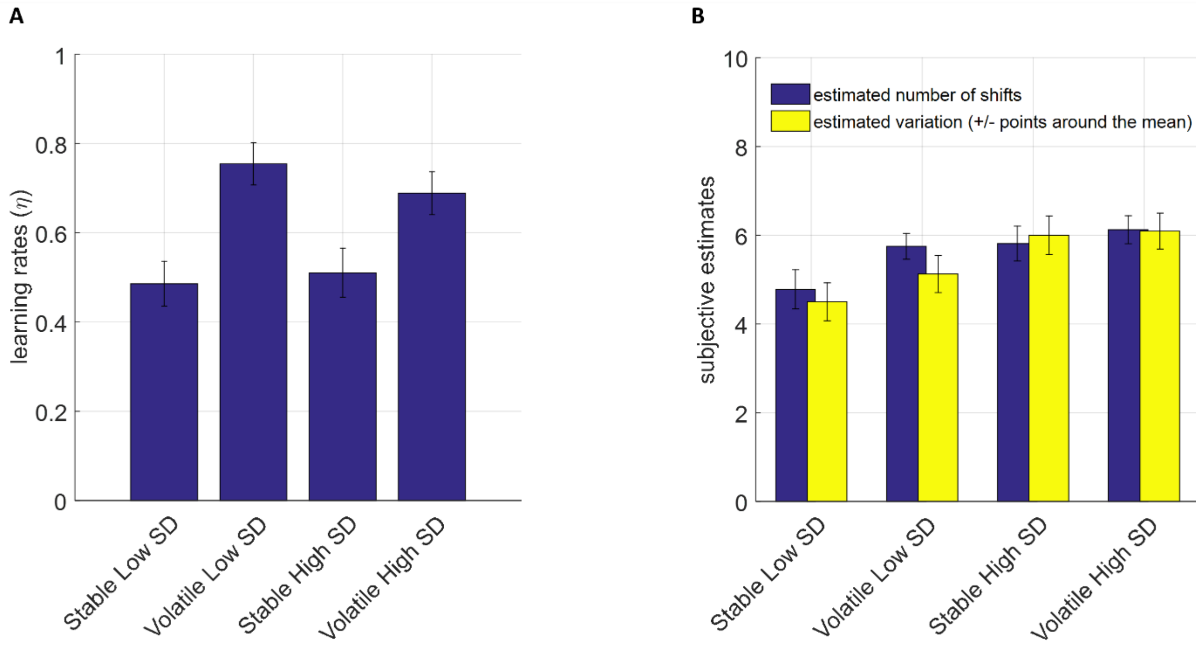

Supplementary Figure F. Behavioural results. (i) Learning rates measured by the RW model fitted to each block separately, demonstrate an overall normative behaviour in human learners[3]. **(ii)** Participants' self-evaluation of environmental volatility and noise did not reflect the changes in the task structure. Estimated number of shifts aiming to capture volatility in the environment, whereas variation around the mean aimed at capturing participants' inference about environmental noise.

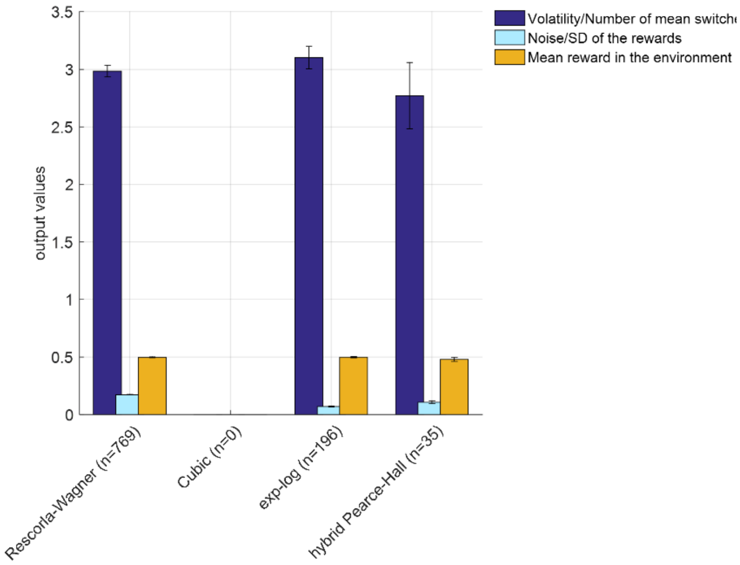

Supplementary Figure G. The results of simulations highlighting the descriptive features of environments which favour one model over the others. In roughly 20% of the randomly generated dynamically changing environments, the exponential logarithmic model can show adaptation more successfully, and these environments were associated with not significantly higher volatility, but significantly lower environmental noise.

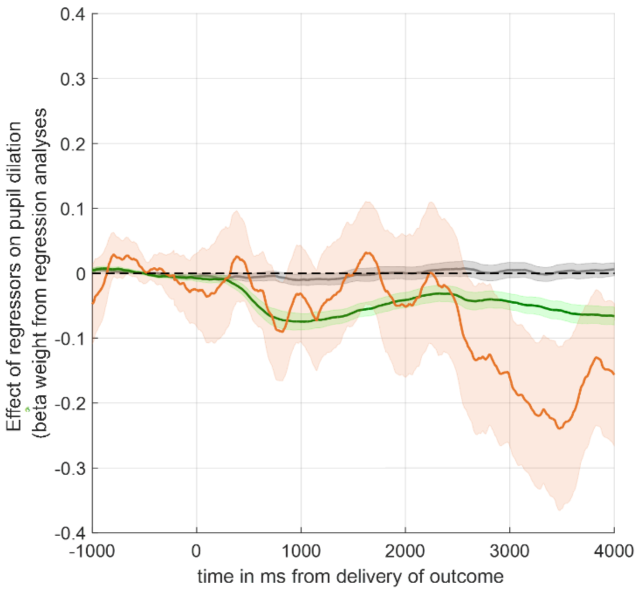

d

Supplementary Figure H. Physiological correlates of learning rates (red line with corresponding error shading) under the Pearce-Hall model. Error shading designate ±1 SEM.

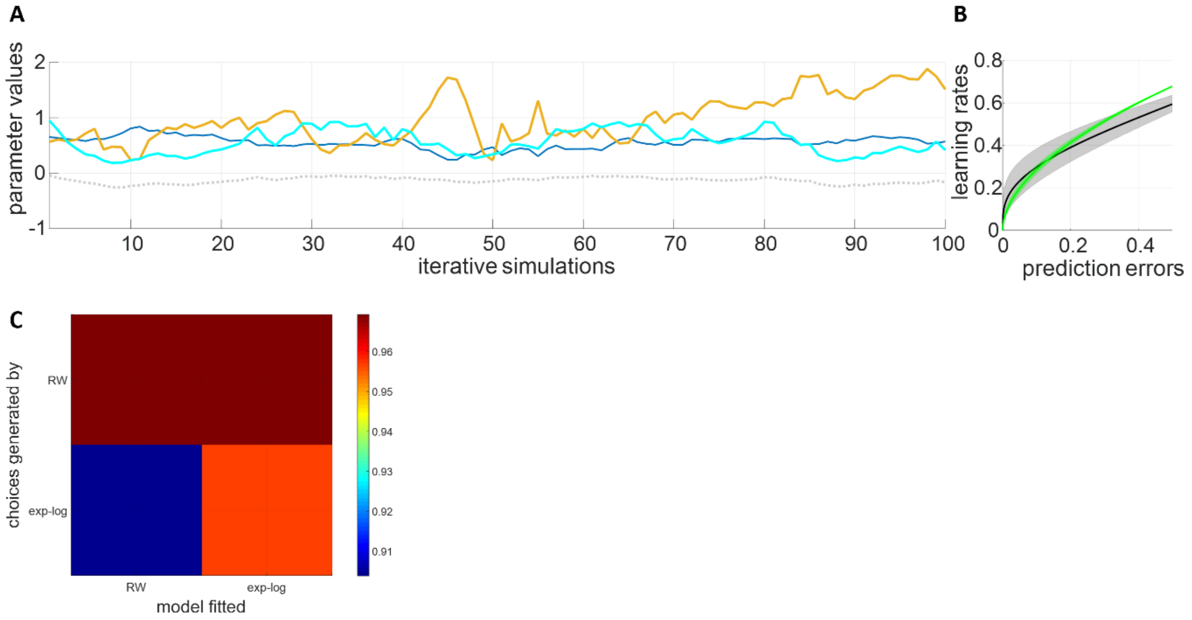

Supplementary Figure I. Generate-Recover simulation results. (i) Initiating from population mean parameter values of the exponential-logarithmic model (delta: cyan; gamma: navy; beta: orange line) in a randomly selected task environment, 100 iterative stochastic generate recover procedures were conducted (i.e. choices were generated stochastically based on initial parameter values and parameters were re-estimated based on newly generated choices). If a deterministic procedure is used (e.g., chose left if choice probability for the left shape is higher than 0.5) the parameter estimates would flat-line from the third simulation onward. Log likelihood of the model (dashed grey line at the bottom) remains at a very desirable range throughout 100 simulations. Beta is linearly transformed by dividing the values by 100 for plotting purposes (thin navy line). Orange and cyan lines designate the gamma and delta parameters of the exponential-logarithmic model. Data stochastically generated by the exponential-logarithmic model is better explained by this model rather than the RW model (means of predictive accuracy based on stochastically generated choices and out of 240 trials in the original task environment: 88.4% vs 80.4%, $t(31)=20.478$, $p<.001$). **(ii)** The relationship between learning rates and prediction errors plotted (green line and error shading) based on the mean parameter values from the generate-recover simulations shown in panel A. Black line and grey error shading denotes the population mean of the behavioural experimental results reported in the main body of the article (Figure 8A). **(iii)** Results of generate recover simulations directly comparing the RW and exp-log models. Choices were generated by relying on the population mean of parameters as obtained from the main analysis, RW=[.5992 5.8259], exp-log=[.6723, .7045 & 5.8987]. After the generation of choices, the models were re-fitted and model probability evaluated. The confusion grid suggests that when choices were generated by the RW model, both models fit the data equally well, whereas when the choices were generated by the exp-log model, exp-log model fits the data better. Colour bar shows the likelihood of the model accounting for the synthetically generated choice data.

References:

1 Pulcu, E. & Browning, M. The misestimation of uncertainty in affective disorders. *Trends in Cognitive Sciences* (2019).

2 Pulcu, E. *et al.* Using a generative model of affect to characterize affective variability and its response to treatment in bipolar disorder. *Proceedings of the National Academy of Sciences* **119**, e2202983119 (2022).

3 Pulcu, E. & Browning, M. Humans adapt rationally to approximate estimates of uncertainty. *bioRxiv*, 2023.2011. 2026.568699 (2023).

4 Nassar, M. R. *et al.* Age differences in learning emerge from an insufficient representation of uncertainty in older adults. *Nature Communications* **7**, 11609 (2016).

5 Nassar, M. R. *et al.* Rational regulation of learning dynamics by pupil-linked arousal systems. *Nature neuroscience* **15**, 1040-1046 (2012).
