# Supplementary material for: A nonlinear relationship between prediction errors and learning rates in human reinforcement-learning
Source: PLoS Comput Biol. 2025 Sep 12;21(9):e1013445. doi: 10.1371/journal.pcbi.1013445 (PMC12449023; doi:10.1371/journal.pcbi.1013445)
Supplement: Attachment — Submitted filename: PLOS_CB_review_comments.docx [file pcbi.1013445.s003.docx]

**[Response to reviewers are typed in bold]**

Reviewer's Responses to Questions

<b>Comments to the Authors: <br/>Please note here if the review is uploaded as an attachment.</b>

Reviewer #1: Ikwunne et al. focused on the potential non-linear relationship between learning rate (LR) and prediction error (PE), and proposed an exponential-logarithmic function that is then tested with (a) simulation, (b) existing data, (c) new experiment.

The idea of looking into the relationship between LR and PE is novel and the paper is generally well-written. However, I have some concerns regarding the analytical approach. I list them below (alongside other points) and hopefully they are helpful.

**Thank you very much for encouraging remarks. In this iteration we made substantial amount of improvements to the manuscript in response to reviewer feedback.**

(1) I would not use the symbol theta for describing outcomes (Eq1). This is because Greek letters are usually used for free parameters (like the eta for learning rate here). So perhaps call it O for the outcome.

**This is revised as the reviewer suggested.**

(2) I generally disagree with the approach of fitting the same model separately for each block (eg, Line 82; see also comments # below ), unless the change of reward contingency is explicitly announced to the participants. The idea of using computational models is to approximate the cognitive process, i.e., the model knows that the participants know, and acts as if the model is a human participant. Now that in the current design (including the authors' previous work) participants were not aware of the clear boundary of blocks, how is it plausible to assume the model knows the blocks and then fitted separately? Indeed, the authors have existing work published using this approach, but this does not necessarily mean the approach is valid. A more proper approach, however, is to fit the model to the entire data across all blocks. In fact, there is the advantage of allowing for a dynamic learning rate because this will be able to capture participants’ behavioural adjustment. Then, one can analyse the learning rates corresponding to each block. This approach is fundamentally different from the current approach in the paper, in that, the model remains agnostic to the block structure, same as the participants. This general principle should be applied across all analyses: simulation, existing data, and new experimental data.

**This was an interesting angle to approach the data and one which we haven’t thought about, and can definitely understand the merits of it. We provided this analysis additionally for Behrens data as we were not sure of whether the task at any breaks or whether the beginning or end of each block were informed to the participants. This analysis did not indicate any meaningful differences between the models and the results are written in detail on page 14.**

(3) Some literature is missing when discussing the types of uncertainty and the interpretation of the learning rate. E.g., Piray & Daw (2020; 2021), Zhang et al., (2020), Rmus et al., (2023). Relatedly, some claims are not 100% accurate. Eg, Line 85, a stable environment does not necessarily mean that the contingency always stays the same; it could well be an experiment with 160 trials and one and only one reversal halfway.

**We have revised the section is highlighted by the reviewer and referenced the recommended papers.**

(4) Line 136, how is this “denial” example related to the current context? If the agent is always denial, then there should always be a -1 PE; but isn’t it the case, according to the current proposal LR-PE model, that when PE is high, the learning rate should be high as well? I find this example confusing.
**In the light of reviewer feedback, we revised the section and removed the example framed around denial in the face of outcomes.**

(5) For the parabolic model (Eq4), I understand that the “4” in “4k” is to guarantee that the focal point is (0,k). But it would be helpful to explain why a focal point is important to have, in addition to being an important mathematical property.

**Here, we highlighted that the focal point of the parabolic curve adjusts the steepness of the relationship between prediction errors and learning rates.**

(6) I do not fully understand Eq6 and the associated arguments. If “LRs decay exponentially in noisy”, shouldn’t there be a negation sign in front of X in Eq6? And what does X mean exactly? It seems to be some sort of predefined constant, but X is usually the “changing variable”. So this reads rather confusing.

**We now provided more clarity on the exponential relationship between uncertainty and parameter K, which is also shown in supplementary figure 1.**

(7) Regarding the side-by-side comparisons of all models with simulation (Fig4):
 - For simulation, it’s great that exact parameters are reported (Line 242-245), but I assume a softmax function was used for the simulation? Softmax and the related parameter was never introduced.

**Just to clarify we did not use a softmax function for the simulations as it would require making different assumptions about the choice stochasticity in addition to the learning process.**

 - More crucially, how exactly was data simulated? The statement “LRs were estimated under each RL model by minimising the average PE trial throughout the task environment” does not seem common to me, as the usual procedure is to minimize the (log) likelihood of choice (via maximum likelihood estimation, LME). How is minimizing PE related to LME?

**In the simulations, our main focus was to understand whether an agent using any of these models have a chance of making adjustments to the environment near optimally, so the key focus becomes whether the model can actually produce outcomes which converge to what is available in the environment. This is why we framed this similar to a maximum likelihood approach.**

 - More importantly, I am lacking a qualitative comparison between the parabolic and the double-weighting model – what do they mean cognitively and how do they differ? Some intuition and examples will be helpful.

**We have now added that parameter K cognitively accounts for individual variability in how steeply learning rates should increase in the face of prediction errors. On the other hand, exponential logarithmic model is a more flexible version of the cubic model and there it allows participants to have different kinds of sensitivities (i.e. ramping up and slowing down in different prediction error ranges) to increase learning rates in the face of prediction errors.**

 - Related to my comment #2, were simulations done per block separately? It shouldn’t given the reasoning I provided.

**We completely agree with the reviewer’s reasoning and the simulations were not analysed block wise as it was conceptualised as a continuous learning environment that changes in time.**
 - Line 250, Fig S2. The relationship between LR and PE for the Pearson-Hall model can also be plotted.

**We included the learning rates coming from the Pearce-Hall model in the supplementary figure along with the other models.**

 - More formally, model comparison is needed to compare the 4 models with simulated data. In fact, since data can be simulated from all 4 models, a model recovery is necessary to see if the predictions of the 4 models can really be distinguished.

**In the current iteration of the manuscript, we provided many different ways of model comparison and wide majority of these comparisons suggests that models cannot be reliably differentiated across different learning/task scenarios. As a result, we emphasise that the novel models that we propose are not superior but viable alternatives with added benefits of investigating the relationship between learning rates and prediction errors.**

 - Also, it would be helpful to use a different colour scheme. The current curves in Fig4 are difficult to read.

**Because the model performance is reasonably close, it remains difficult to differentiate these models. Therefore we relied on bar figures (e.g. supplementary figure 2) and trial timeline figures (e.g. supplementary figure 4) where it is possible to differentiate the models.**

(8) Sections of Human Data:
 - related to my comment #2, are the blocks shown to the participants (line 277, line 350)?

**In our own experiment, the blocks were shown to the participant separately and this is now clarified in the text, please see section from line 454**
 - line 286, what is the equation of the “risk attitude”? It is not properly introduced.

**This was modelled by a power utility parameter commonly used in Prospect Theory**
 - why only show the relationship between LR and PE from the double-weighting model? How does the relationship look like for other models?

**We apologise for the omission and included other models in this analysis now**
 - line 298, I don’t think analysing log likelihood with ANOVA is a good approach for model comparison, as LL does not yet consider model complexity. Information criteria such as AIC is required.

**In the additional analysis we included information related to model AICs as well as Bayesian Omnibus risk which gives a metric of how likely it is that models cannot be reliably differentiated.**
 - I am not sure what the purpose is for including pupil data? How exactly is it closely related to the main argument the current study is trying to make? It is not necessary if it is only a “good to have”.
**In the current submission we try to highlight that the pupillometry allows us to test a temporal prediction about the learning rates coming from the novel models and we provided additional analysis of the pupilometry data with the Pearce-Hall models to show differences between results. Our reasoning in collecting pupillometry data was to support the viability of the novel RL models with additional physiological support.**

Reference:
Piray, P., & Daw, N. D. (2020). A simple model for learning in volatile environments. PLoS computational biology, 16(7), e1007963.
Piray, P., & Daw, N. D. (2021). A model for learning based on the joint estimation of stochasticity and volatility. Nature communications, 12(1), 6587.
Zhang, L., Lengersdorff, L., Mikus, N., Gläscher, J., & Lamm, C. (2020). Using reinforcement learning models in social neuroscience: frameworks, pitfalls and suggestions of best practices. Social Cognitive and Affective Neuroscience, 15(6), 695-707.
Rmus, M., Zou, A., & Collins, A. G. (2023). Choice Type Impacts Human Reinforcement Learning. Journal of Cognitive Neuroscience, 35(2), 314-330.

**We reference to all of these papers recommended by the reviewer and we would like to thank the reviewer again for all the constructive feedback which we think improved our paper from its original submission.**

Reviewer #2: In this study, the authors propose two novel adaptations of the Rescorla-Wagner model of reinforcement learning in which the learning rate changes dynamically as a cubic or exponential-logarithmic function of the magnitude of the prediction error. They show, using simulations, re-analysis of published datasets and analysis of original data, that the models are able to account for choice behaviour on a range of learning tasks involving different levels of stochasticity and volatility, while yielding trial-by-trial learning rates. They also present pupillometry data and relate it to the magnitude of prediction errors, learning rates, and outcome magnitude. They conclude that their models are adequate to study learning deficits in mental health conditions.

**We thank the Reviewer for a succinct summary of our paper.**

Although interesting, I do not believe that the paper is ready for publication at this time. The research is not sufficiently motivated: the arguments in the introduction are structured somewhat unintuitively, the research questions are neither explicitly stated nor their significance explained, and the authors formulate no hypotheses, nor do they introduce the methods they use to test them in the introduction.

**We thank the Reviewer for these higher level feedback. In the current iteration, the introduction section is substantially rewritten.**

Moreover, it does not become clear how and why the models the authors propose advance the field: if I understand correctly, neither model has an advantage in fit over existing models, and the properties the authors identify as desirable – that the models yield trial-wise learning rates that are uncorrelated with prediction errors – are also met by a pre-existing model they discuss.

They further disregard a model from the recent literature in which learning rates are similarly adapted to volatility and stochasticity in the environment (Piray & Daw, 2021, Nature
Communications). This should be engaged with. Nonetheless, I believe the manuscript has potential, and with some revisions, it could make a meaningful contribution to the field.

**In the current revised submission we highlighted that our models complement the ones existing in the literature and substantially simpler than Bayesian solutions to the adaptation to different sources of uncertainty.**

I have some specific comments that the authors may find useful as they revise their manuscript:

Major
(1)     The reasoning behind the research does not become sufficiently clear. I would suggest specifically that the authors:
a.      State research aims/questions & explain why they are important
b.      Explain why methods are adequate & motivate experiments (e.g., it does not become clear why pupil dilation is relevant)
c.      State hypotheses
d.      In the discussion, come back to hypotheses and aims

**the revised paper is rewritten in the light of these feedback above.**

e.      Ensure they clearly show the advantage of their novel models above and beyond existing ones
**we demonstrated evidence that our models can reliably estimate learning rates in trialwise manner which cannot be achieved by the Rescorla Wagner model and our analysis of the pupillometry data lends greater support to our proposed model relative to the Pearce Hall model which also fitted to our data worse.**

(2)     Separate introduction from model development, which is part of the results.

**Although we are not 100% sure whether we have achieved this clear-cut separation, we have substantially revised our introduction section in the light of reviewer feedback.**

(3)     Engage with the Piray & Daw model. One option could be to pitch the models described in the paper against the Piray model in empirical data (crucially performing model comparison).
**We have cited all the relevant research from Piray and Daw in the current iteration. We have also evaluated the scripts from their model which was similar to the one published in our original and even earlier paper (Pulcu & Browning, 2019 and subsequently 2022). These Bayesian solutions are substantially more complex than any of the models that we are proposing (both of the models operate over a single equation, rather than many interlinked assumptions). We dedicated substantial amount of space to discuss the Bayesian solutions to the decision-making problem (from line 152) and we had also compared our models to our own Bayesian solution (perhaps this must have been overlooked in the initial submission but see Supplementary Figure 2; it was not possible to implement the scripts from Piray & Daw, due to the complexity of their coding). As we have already shown with our own comparison, the current simple models cannot match the performance of the Bayesian solutions. I think all the reinforcement learning experts would agree that this is simply an unrealistic benchmark to achieve. We hope that the reviewer will appreciate the simplicity provided by the models that we propose.**

**All of the minor points highlighted below are revised and we further sought help with proofreading from our native English colleagues with relevant expertise in reinforcement learning.**

Minor
(1)     The argument in the introduction follows a somewhat unintuitive order. Some suggestions for revision: explain the RL model before discussing learning rates; consider explaining Pearce-Hall after the sources of LR variability; clearly point out limitations before presenting novel solutions.
(2)     Given that there is no word limit, it may be worth it taking the time to explain things in more detail to help readers follow along more easily. Some examples where I thought more explanation would be helpful: l. 73 “In computational neuroscience, LRs are numerically estimated from participant choices by using different RL algorithms -which are in effect approximations of the Bayesian models that can also estimate otherwise hidden generative rules of dynamically changing environments.”; l. 82 “RW model as a measurement tool” – as a measurement tool for what?; l. 103 “smoothing the transition of the probability update” – what does that mean?; l. 135 ff. it would be helpful to list the assumptions (as in “First, …; second…;”; l. 203 in the equation, delta and lambda are not defined and it is unclear how they are to be interpreted; l. 310 the trial sequence, contingencies etc. of the bucket task are not explained in detail; l. 416 it is not clear what is meant by “Signed
PEs encoding the global task structure”; l. 474ff “our choice of 0.5 as average reward rate in stable environments inevitably pushed towards maximum entropy” – this statement is not explained; l. 485 “similar to a recently described neurophysiological reward-dependent metaplasicity model that can account for the learning behaviour of non-human primates” – the model is not explained.
(3)     More precision in some descriptions could help avoid confusion. Examples of imprecise descriptions include: l. 73 “In computational neuroscience, LRs are numerically estimated from participant choices by using different RL algorithms” – they are estimated using optimization algorithms, and are part of RL algorithms, right?; l. 107 “different sources of uncertainty dictate which style of learning …” – speed would be a more precise term, style can be anything, correct?; l. 135 “probabilistic contingencies (i.e. reward versus no reward)” – binary is not the same as probabilistic; l. 173 in “noisy environments with high expected uncertainty” it is not clear what the difference between noisiness and expected uncertainty is; l. 332-333, what “behaviour” does the statistic refer to?; l. 345 mentions fractals but the symbols in Fig. 7 are not fractals.
(4)     Some sentences could use shortening and/or a quick grammar check. Some example sentences I found difficult to follow: l. 65ff “Living in an uncertain world continuously probes learning systems in the brain, guiding agents to make better decisions. This is a type of value-based decision-making is critical for survival in the wild and long-term evolutionary fitness.” – it is not clear what guides agents, and what “this type” of value-based decision making is; l.80 ff “However, a key limitation of the RW model is that it is commonly fitted to participant choices within a block of an experimental task, estimating participants' average LRs during that period. This is an approach that uses the RW model as a measurement tool, implemented by a number of key RL studies consistently showing that human LRs are higher in volatile (i.e. environments in which contingencies change), relative to stable environments in which contingencies stay the same.” – it is not clear what is doing
the “estimating” and what “this approach” is; l. 283:288 “More specifically, participants' decision values were computed by integrating their reward probability estimates (computed under each RL model) with their corresponding reward magnitudes associated with each option (modulated by a single power utility parameter accounting for participants' risk attitude) and calculating the expected value difference between two options and choosing between them stochastically following the choice probabilities generated by a sigmoid function” – this could be broken up into two)
(5)     Figures could be made more accessible and adequately labelled (e.g., Fig. 6c could use a legend)
(6)     There are a few typos (e.g., “This is a type of value-based decision-making is critical”; “metaplasicity” …)

**We would like to thank the reviewer for all the insightful comments provided. We think addressing these improve the quality of our manuscript substantially over its initial submission.**

Reviewer 3:

This manuscript proposes an elegant approach to estimating how reward prediction errors are used to update learning rates in dynamic environments. The authors begin from the assumption (well established by previous work) that adjusting one’s learning rate in response to different levels of volatility and observation noise leads to more optimal inferences about outcomes. They suggest that linear updates, assumed by most prior models with a dynamic learning rate, are not always ideal, and that nonlinear functions can better account for peoples’ tendency to discount prediction errors resulting from noise. A major strength of the proposed alternative update rules is that they are overall quite simple while still capturing this idea. Through a series of simulations and analyses of behavioral tasks, the manuscript demonstrates that at least one of these models explains behavior about as well as prior models. Overall, this is an important topic relevant to many researchers who study reinforcement learning and decision making. Despite the strengths of the work, though, there are several areas where the manuscript can be improved. I have separated my comments into major and minor concerns below:

**We thank the Reviewer for these encouraging feedback and we endeavoured to improve the quality of our manuscript in response to reviewer suggestions in the current iteration.**

**Major comment #1**

The conclusion made in the abstract that, “the relationship between PEs and LRs … can be better accounted for by an exponential-logarithmic function” is not currently fully supported by the current analyses. The primary issue I see with this lies primarily with the model comparisons that were performed throughout the paper. In particular, while four models are simulated (a fixed learning rate Rescorla-Wagner, a hybrid Pearce-Hall which assumes a linear update function, and their two nonlinear learning rate update models), these models are not systematically compared across all of the tasks.

**In the revised submission we systematically tried to improve on this shortcoming and fitted and compared all of the models across all experiments, using different forms of model comparison along with corresponding caveats.**

In re-analysis one (of the Behrens data), all four models were fit and compared using model log-likelihood, but the exact methodology for performing this comparison was not made clear. Because the models differ in their number of free parameters, a model comparison procedure that penalizes models based on their number of free parameters should be performed to better understand which model is best performing. It is reported that there were no differences in fit on this task, but to support the conclusions made in the abstract, one of the two novel models in this paper should outperform both the rescorla wagner and the hybrid pearce-hall model, at the least. Furthermore, it seems important that the models also outperform (or at least match the performance of) the Bayesian model used in the original paper. It seems that the authors have simulated this model too (based on the “Bayesian Ideal Observer” reported in supplementary figure 1), but there isn’t enough information provided to determine whether this is the case.

**We apologise that we probably misled the Reviewer inadvertently. We did not compare the performance of our models to the Bayesian observer which was published in the original Behrens et al paper. We compared our models to our own Bayesian ideal Observer published in (Pulcu and Browning, 2019; Pulcu et al 2022), this is now reported in Supplementary Figure 2. Our own Bayesian solution essentially uses the same architecture proposed by Behrens et al, but instead of only dealing with environmental volatility, it can also deal with environmental noise. As we have explained in our response to Reviewer 2, Bayesian solutions are far more complex with multiple assumptions about the nature of the relationships between uncertainty and the learning processes, therefore pose an unrealistic benchmark for these simpler update rules to excel. As we have already published our own Bayesian solution to the learning problem some time ago, the current paper deals with a different question and does not attempt to provide yet another Bayesian solution that outperforms all the other complex models in the literature.**

In re-analysis two (of the Vaghi data), only the two novel models are compared to one another. All of the models should be compared on this task as well in order to fully support the idea that the proposed nonlinear relationship between PEs and LRs indeed better accounts for participants’ behavior. Further, given that models were not penalized based on their number of free parameters and the exp-log model (with 2 free parameters) outperforms the parabolic model (with only 1), it is currently difficult to determine which of their two models is actually best on this task.

**In the revised submission we compared many of these models to give the full picture and in our own evaluation the exponential logarithmic model fits the data the best.**

Finally, regarding the analysis of the new data provided in this study, only the exp-log model was compared to a RW model. It seems critical that at least a PH model be compared here as well (in order to rule out linear LR updating) and the authors’ stated reason for focusing on only the exp-log model does not justify this limited comparison given that predictions of choice behavior are generated by all of the models. I also think that including a comparison of the RW model with a single learning rate to the reported (supplementary figure 5) RW model with multiple learning rates is an important baseline for this task as well, given that participants reported no subjective differences in volatility or noise (lines 368-370; supplementary fig 5B). If people did not alter their learning rates at all in this task, it seems unlikely that a dynamic learning rate model would provide a better fit. In the discussion the conclusions are tempered “newly proposed models perform comparably well with the existing RL models” (lines 431-432) relative to the abstract, but I think even this statement cannot be fully supported without performing the above comparisons and penalizing free parameters.

**Here, we would like to highlight that indeed our participants altered their learning rates in relation to environmental noise and volatility, it was only for stable outcomes the level of noise did not need to significant differences in learning rates as the underlying outcome schedule remains globally uninformative (Supplementary Figure 6A). Overall, we think a conservative evaluation of our models across different tasks is that they perform comparably to the existing learning algorithms and generate trialwise learning rates which can be used in neuroimaging analysis. We think, having these models will give cognitive neuroscientists and clinicians more options to analyse and interrogate the relationship between prediction errors and learning rates in different conditions, therefore we think they would provide a benefit to the RL literature and catalyse an interest for the relationship between learning rates and prediction errors which is often/frequently neglected.**

**Major comment #2**

My second major comment is that the authors do not adequately cover recent developments in Bayesian and statistical estimation models that solve similar problems to the proposed methods. There are brief mentions to the original Behrens ideal observer, the hierarchical Gaussian filter (line 86), and the reduced Bayesian model developed in Nassar et al., 2010 (lines 179-182). But, notably, several papers (Piray & Daw, 2020; Piray & Daw, 2021, for example) have also explored how Kalman filtering can be used to estimate outcomes and dynamically update learning rates. Importantly, Kalman filtering also reduces to a RW-style update, similarly to the authors’ log-exp model. I think to fully demonstrate that either of the authors’ nonlinear LR update models improve upon past work, at least one model from this general class should be included in the model comparisons. Relatedly, the authors motivate their models in multiple places (lines 128-130, lines 164-169, for example) by contrasting PEs resulting from either unexpected or expected uncertainty. However, disambiguating the source of PEs is not as straightforward a problem as the writing would suggest here. For example, if the expected level of uncertainty is unknown (e.g. at the beginning of learning), it is very unclear what could have caused a large PE. Recent Kalman filtering models have been developed to solve this problem (Piray & Daw, 2021), and so it would be particularly useful to demonstrate that the authors’ model(s) could outperform methods crafted for this purpose. Code is available online for practically all of the above mentioned models, so hopefully adding at least one to the comparison would not be too much trouble. Generally, I think that the paper and overall contribution to the literature would be strengthened substantially if the authors could demonstrate that either of their models (which are indeed quite simple, elegant, and intuitive) outperform or perform similarly to these much more complex models (some of which are likely cognitive unrealistic).

**As expressed in response to the Reviewer 2, we have dedicated a section to review the literature on Bayesian solutions to the adaptations to different sources of uncertainty, citing all of the relevant literature also highlighted by the reviewer. However, we were not able to implement the model from Piray and Daw as it was not possible to understand the flow of their coding even if the source code was readily available. I am sure many people might think something similar to our own Bayesian solution to the decision problem as reported in our earlier papers where the code is also openly available. As we have shown in our own analysis, these simpler update rules can never match the performance achieved by complex Bayesian models and we think this is a rather unrealistic benchmark to meet. We completely agree with the reviewer’s interpretation that, these mostly grid-based Bayesian models propose an unrealistic structure with which reinforcement learning is implemented cognitively and computationally in the human brain. We explicitly highlight that our paper is not attempting to come up with a single model which outperforms all the other complex solutions reported in the existing literature. Personally, I think coming up with a very simple update rule which can outperform all the existing advances in Bayesian modelling will be impossible as it will mean that the whole field has been missing something which was so obvious for so long. We believe that we still provide novel, simple and elegant update rules (also kindly praised by the reviewer) which we hope will stimulate further research and interest in the relationship between learning rates and prediction errors.**

**Major comment #3**

**We have made substantial improvements to address all of the points raised by the reviewer below.**

The manuscript would benefit greatly from careful (line-by-line) editing for mistakes throughout and close proofreading during revision. Furthermore, the writing and organization are unclear in several places, making it somewhat difficult to follow. A few examples (although I did not track all of them):

- The point being made by sentence 2 (lines 65 - 67) of the introduction was not clear to me

- A typo on line 67: “this is a type of…” should be “this type of”

- In figure caption 1: line 153: “was relative to..” should be “were relative to..”

- Throughout the figures, the cubic model is referred to as parabolic (this should be made consistent throughout)

- Figure 5B and Figure 6B are not referred to in the main text of the results

- On lines 119-124, a more clear distinction should be made between uncertainty resulting from surprise and uncertainty resulting from the expected level of volatility/hazard rate of an environment. The description of expected uncertainty is clear, but from the description of unexpected uncertainty it isn’t clear which of these the authors are referring to. These concepts are easily confusable and it would help the authors communicate to the reader what aspects they are addressing directly if they made this distinction more clearly.

- I believe that some additional handholding throughout would also help the authors communicate their findings. In particular, including a paragraph at the end of the introduction leading into the results would help to make clear what the purpose of the analyses are, and what the hypothesis is more generally. At this stage in my reading, I was not clear on whether the goal was to test whether the cubic model was best and why the data-driven approach was being introduced.

- At the beginning of the results, the purpose of the simulations was also unclear. This was eventually revealed (to demonstrate that their models perform on-par with traditional models) but should be laid out prior to presenting the simulations.

- Similarly, at the end of the discussion, the conclusions that we should ultimately draw from the work were not obvious. Which model do the authors believe participants are using, and how much does this depend on the particular task? If the log-exp model does indeed better explain the data and captures the cubic model as a special case, why is there such a strong focus on the cubic model? I think that all of these points are touched upon throughout the discussion, but they relate to my earlier methodological concerns, so I believe it will be easier to draw clear conclusions from the study once more systematic model comparisons have been performed.

**Minor comments**

- On line 360, the authors say that supplementary figure 5A shows evidence of near-optimality based on RW model fits, but it is not clear how this shows optimality (I assume the authors mean that learning rates pan out according to what would be expected for noise/volatility). But it would be helpful to show what the “optimal” learning rates are here to compare to in order to warrant this claim.

**Here, we have referenced our recent work which utilises the same Bayesian ideal Observer model that we published before (Pulcu & Browning, 2019; Pulcu et al., 2022) and there we showed that Bayesian model displays behavioural adjustments that is reasonably overlapping with our participants as now reported in Supplementary Figure 6A, therefore we kept the near optimal language as before.**

- Regarding the pupillometry analyses, I believe a stronger test of whether LRs are updated on the same trial (as predicted by the log-exp model) or not (as predicted by pearce-hall) would be to also compare the effects of Pearce-Hall learning rates on pupil size to the LRs extracted from the log-exp model. More broadly, though, given the potential confound of increased luminance during the outcome presentation period, I am not sure how much these results add in general. They may be better relegated to the supplementary information.

**We really appreciate the suggestion from the reviewer and in the current revised submission, we have also analysed the pupillometry data with Pearce Hall model, and this analysis seems to better support our proposed model then PH.**

- On line 465, it doesn’t seem relevant to mention funding limitations, as there is no need to apologize for not including neuroimaging. Instead it would be better just to say that neuroimaging should be addressed in the future.

**We wanted to highlight this limitation as functional neuroimaging can provide a very rich insight as to the implementation of reinforcement learning in the human brain and we hope that this paper will catalyse a further interest in this question, particularly the relationship between learning rates and prediction errors in the human brain (as previously proposed by a handful of studies).**

**Comments on the figures and figure captions**

- Figure Caption 2: there is much discussion about K, but the relationship between K and sigma is not shown in the figure at all. I understand that this is relevant to the overall point of why the relationship between sigma and LR is being shown here, but this is confusing in its current presentation. The authors should consider including this information in the main text instead.

**We thank the reviewer for flagging this up and this is now included as Supplementary Figure 1.**

- Figure 3 is difficult to read because the lines are not clearly mapped on to values of the parameters. It would be more clear if some lines were highlighted and the parameters that produced them were shown alongside.

**We appreciate the feedback from the Reviewer. Because the exponential logarithmic function parameters interact with each other to generate different trajectories, it is not very straightforward to map the trajectories onto individual values as they interact in non-linear ways. Below we show an example of what happens if (i) values of gamma and Delta are identical; (ii) values of gamma is larger than Delta; (iii) values of Delta larger than 2x gamma**

**note that here we also needed to make the prediction error steps more coarse for plotting purposes. To improve clarity and transparency, we decided to share this script which can allow the readers to play around with different parameter ranges and steps to visualise their interactions.**

- Figure 4: The authors should consider plotting the lines on different plots, or adjusting opacity or line thickness, because it is difficult to distinguish estimates from individual models in the current figure.

**Although we principally agree with the reviewer in terms of visualisation of different models, because they perform ever so closely, separating the lines into different panels did not provide any further clarity as each panel ended up looking pretty much identical. This is why we reference to supplementary figure 2 where the slight differences between the models (mostly non-significant) are visualised across bar plots.**

- Figure 6C: It isn’t clear what the line/band is, or which model it is from. I assume this is the from the exp-log model, but this should be made more obvious.

**We apologise for the omission. The reviewer is correct and this is now highlighted in the Legends to the figure.**

- Figure 7: The same outcome screen appears twice at the end of the trial sequence. I was not sure whether this was a mistake or intentional. If the latter, this should be made clear.

**We appreciate the reviewer for detecting this mistake, which is now corrected in the revised submission.**

- Figure 8B: The authors should include a legend for the different lines in the actual figure

**in the current iteration, we provided the legend to the lines in the text, fearing that adding further text might clutter the figure.**

**We thank the reviewer for all the insightful feedback which helped us to improve the quality of our manuscript substantially over its initial submission.**
