# Supplementary material for: A nonlinear relationship between prediction errors and learning rates in human reinforcement-learning
Source: PLoS Comput Biol. 2025 Sep 12;21(9):e1013445. doi: 10.1371/journal.pcbi.1013445 (PMC12449023; doi:10.1371/journal.pcbi.1013445)
Supplement: Attachment — Submitted filename: Ikwunne_PLOSCompBio_2.docx [file pcbi.1013445.s004.docx]

While I appreciate the authors’ efforts in responding to my previous comments, I am still reluctant to recommend the paper for publication. My main problem is that the authors still do not clearly demonstrate that their new models advance the field – all they show is that they do not do worse than existing models (using unusual model comparison methods). This is insufficient. Either the authors can show that there are contexts in which their models outperform existing ones, or else they need to change the main message of the paper to “Elegant RL model that can dynamically adapt to expected and unexpected uncertainty fails to improve model fit in simulations and human choice data”. The latter would also be a valuable contribution, however, the manuscript would have to be substantially rewritten. Beyond this, I have some further suggestions which I detail below and hope the authors will find helpful.

(1) Introduction
    a. The authors refer to a “knowledge gap” with regard to the relationship between LRs and PEs. But as I understand it, LRs and PEs are not things in the world whose relationship can be studied empirically and thus learned about. Instead, they are model components whose relationship is *defined* by the models themselves. The authors seem to agree, since they formulate models to accommodate *theoretical* assumptions about that relationship. So the statement of the research aim as “describing simple RL algorithms that can adequately address this knowledge gap” is confusing to me. Perhaps the situation is better described by saying that there’s a knowledge gap with regard to how people adapt their learning rates to changes in expected and unexpected uncertainty in the environment, including neural correlates and interindividual differences? And the research aim would be to provide a simple RL algorithm that allows for flexible adaptation of LRs to both (while avoiding the admittedly forbidding complexity of Bayesian models)?
    b. In general, I think that the introduction is unnecessarily broad and long and could be streamlined. A short, crisp introduction could follow – for example – an outline like this:
       i. RL models are widely used to explain learning and decision-making in various environments.
       ii. Common models (RW, hybrid PH) are inadequate in capturing dynamic changes in expected and unexpected uncertainty.
           1. Explicitly state theoretical assumptions regarding:
              a. Unexpected uncertainty
              b. Expected uncertainty
       iii. Bayesian models, while powerful, are complex and challenging to implement.
       iv. Statement of research aim: To develop simple RL models that account for dynamic LR adaptations to facilitate research into neural correlates and interindividual differences
    c. As mentioned in my previous review, I would move the model formulations to the results section because they are the main contribution of the paper.
(2) Results
    a. I find it hard to get an intuitive grasp of the exponential-logarithmic model. How does it react to different levels of surprise (absolute PEs) and noise (sigma)? What is the role of delta and lambda? As free parameters, how are they to be interpreted conceptually / at a cognitive level? In Fig.3, I can’t make out what the different values of delta and lambda are. Are they shown? If not, why not?
    b. Details on the simulations are missing (number of simulations, parameters etc.) – as it is, I could not reproduce them. If full details take up too much space, this could go to the methods.
    c. As mentioned above, my main issue with this paper is that the authors do not show that their model is superior to existing models. But given theoretical assumptions, previous work, and the simplicity and elegance of their (exponential-logarithmic) model, it is actually surprising (to me) that it isn’t. I would encourage the authors to
       i. try to find a simulated environment where their models do outperform the others, and/or
       ii. attempt an explanation as to why they do not in the existing synthetic and real datasets, and why some of the theoretical assumptions about how LR and PE should be related do not seem to hold. E.g., Fig. 8 seems to show very little adaptation of LR to PEs but rather strong interindividual variance. Likewise, in the same experiment, environmental noise did not influence LRs in stable environments. In addition, volatility did not influence LRs in the Behrens data. All of this is contrary to assumptions.
    d. I still find the model comparison procedure idiosyncratic and somewhat confusing. I would suggest that the authors make a reasoned choice for one single metric (that penalizes model complexity) and stick to it throughout the manuscript.
    e. I would like to see both model and parameter recovery for all models (though specifically for the two new models) (this may also help in answering 2.c).
    f. I encourage the authors to review their figure legends. It seems to me that they sometimes contain much more than descriptions and necessary explanations for the figure (e.g., Fig.2 line 235ff.). Perhaps they can move the rest to the main text, or if not relevant, omit it.
