# Supplementary material for: A nonlinear relationship between prediction errors and learning rates in human reinforcement-learning
Source: PLoS Comput Biol. 2025 Sep 12;21(9):e1013445. doi: 10.1371/journal.pcbi.1013445 (PMC12449023; doi:10.1371/journal.pcbi.1013445)
Supplement: Attachment — Submitted filename: PloSCB_R2_Responses to Reviewers.docx [file pcbi.1013445.s005.docx]

**[Responses to authors provided in Bold]**

Reviewer's Responses to Questions

**Comments to the Authors:
Please note that one of the reviews is uploaded as an attachment.**

Reviewer #1: The authors have done a good job addressing most of my comments, and I believe that the paper is now stronger. I have a few remaining minor points:
**We appreciate the positive feedback from the Reviewer and endeavoured to make further revisions based on their recommendations**.
- Regarding model comparisons, I appreciate that the authors have more consistently compared all models on all of the datasets, and I believe that this allows for much stronger conclusions to be drawn. Yet the cubic model is not compared on the third dataset, despite it being the only model that demonstrates higher performance than all other models on at least one metric (on the Behrens data, via AIC the cubic model is slightly better than the 'runner-up' pearce-hall model and at least somewhat better than the other models, although these values are not reported; on the Vaghi data, of their models the exp-log is best but it performs equally to rescorla-wagner). Given this, I don't think it is fully justified to exclude this model from the comparisons on the final dataset, so I believe it should be included here, both for completeness/consistency but also because the cubic model outperformed the exp-log model on the Behrens data (when fit continuously across blocks).
**We apologise for the omissions. In the revised submission we also reported the AIC values for the competing models in the Vaghi dataset. In our previous submission, we thought because the cubic model did not behave competitively in the Vaghi dataset, we could remove that from our in-lab experiment with a process of elimination as the nature of the learning process was closer to the Vaghi experiment rather than Behrens experiment. In this iteration, we also fitted the cubic model to our own data and we report that its fitting metrics were the worst among all the models. This is in line with the learning rate trajectories that we reported in Figure 8A, i.e. a parabolic curve cannot capture a plateauing exponential curve. Nevertheless, we hope that reporting these details improved the manuscript and provided more information for the readers.**
- On line 398: It is stated that cubic model has three free parameters, including the learning rate, but isn't the learning rate determined by equation 4, which depends on the free parameter kappa, making it not a free parameter?

**The Reviewer is correct, this section is revised to provide clarification.**

- The cubic model is still referred to as the "Parabolic" model throughout the figures--this should be changed to match the text in order to be more clear.

**The Reviewer is absolutely correct, and we apologise for overlooking those instances. The figure legends are now carefully checked and revised accordingly, along with the rest of the manuscript.**

Reviewer #2

While I appreciate the authors’ efforts in responding to my previous comments, I am still reluctant to recommend the paper for publication. My main problem is that the authors still do not clearly demonstrate that their new models advance the field – all they show is that they do not do worse than existing models (using unusual model comparison methods). This is insufficient. Either the authors can show that there are contexts in which their models outperform existing ones, or else they need to change the main message of the paper to “Elegant RL model that can dynamically adapt to expected and unexpected uncertainty fails to improve model fit in simulations and human choice data”. The latter would also be a valuable contribution, however, the manuscript would have to be substantially rewritten. Beyond this, I have some further suggestions which I detail below and hope the authors will find helpful.

**We thank the Reviewer for the constructive comments. In the revised submission, we reported AIC values for competing models and these comparisons suggest that our models perform either ever so slightly better or worse depending on the dataset. They perform consistently better than the PH model which is the only other alternative to extract trialwise learning rates (if we agree that a constant learning rate assumption inherent in the RW model is not correct, i.e. learning rates change when uncertainty is manipulated experimentally). As a result, we were not sure whether using “fails to improve” language was necessary, or a substantial rewrite. However we did go through the manuscript diligently to make sure that we are not over-interpreting or positively biased in favour of our results.**

**Below, we hope that the Reviewer will see that we have made a lot of very targeted improvements to the manuscript based on their suggestions while keeping it closer to how we, as the authors, think it should be, i.e. communicating details and properties of these models to stimulate discussion in the field. We hope that the Reviewer will agree that this paper can achieve its purpose better in PloS Computational Biology [than later elsewhere].**

(1) Introduction

a. The authors refer to a “knowledge gap” with regard to the relationship between LRs and PEs. But as I understand it, LRs and PEs are not things in the world whose relationship can be studied empirically and thus learned about. Instead, they are model components whose relationship is *defined* by the models themselves. The authors seem to agree, since they formulate models to accommodate *theoretical* assumptions about that relationship. So the statement of the research aim as “describing simple RL algorithms that can adequately address this knowledge gap” is confusing to me. Perhaps the situation is better described by saying that there’s a knowledge gap with regard to how people adapt their learning rates to changes in expected and unexpected uncertainty in the environment, including neural correlates and interindividual differences? And the research aim would be to provide a simple RL algorithm that allows for flexible adaptation of LRs to both (while avoiding the admittedly forbidding complexity of Bayesian models)?

**We agree with the Reviewer that learning rates and prediction errors are latent processes which cannot be observed directly. The Reviewer is correct that our current paper relates to investigating the relationship between learning rates and prediction errors and describing models with a priori assumptions (e.g. that this relationship is parabolic) or without any a priori assumption (e.g. using an exponential logarithmic function to reveal the shape of this relationship from data). We do not think reframing this paper around a knowledge gap with regard to how people adjust their learning rates to changes in expected and unexpected uncertainty is a sensible direction. As someone who actively published some key papers in that realm (Pulcu & Browning, 2017, 2019, 2025), I [corresponding author] personally find that space highly saturated, and to me there is not really a knowledge gap anymore, also after key contributions from researchers like Behrens, Mathys, Lawson, Gillan, Nassar, Piray, Daw, (the list can go on…) [perhaps until Deepmind etc. arrives to provide one models that rules over all].**

**From my perspective, providing as much as information as possible about simple and relatable models such as the ones that we propose will be a much more valuable contribution to this field. I really hope that the Reviewer will sympathise with our perspective.**

b. In general, I think that the introduction is unnecessarily broad and long and could be streamlined.

**We really do hear the Reviewer’s feedback. However, we think that this broad introduction is needed as many related concepts need to be discussed (expected and unexpected uncertainty) to be able to explain concepts related to some of the other datasets that we analysed. Below, we are trying to illustrate that our current introduction is not too far away from what the Reviewer proposed.**

A short, crisp introduction could follow – for example – an outline like this:

i. RL models are widely used to explain learning and decision-making in various environments **[in our paragraph 1]**.

ii. Common models (RW, hybrid PH) are inadequate in capturing dynamic changes in expected and unexpected uncertainty **[from paragraph 3]**.

1. Explicitly state theoretical assumptions regarding: **[from paragraph 4 depending on how one counts sections broken by equations related to RW and PH models]**

a. Unexpected uncertainty

b. Expected uncertainty

iii. Bayesian models, while powerful, are complex and challenging to implement. **[Paragraph 5]**

iv. Statement of research aim: To develop simple RL models that account for dynamic LR adaptations to facilitate research into neural correlates and interindividual differences **[also in paragraph 5]**

c. As mentioned in my previous review, I would move the model formulations to the results section because they are the main contribution of the paper.

**In line with the Reviewer’s feedback, we now moved the Results subheading above the section where the cubic model is introduced. We hope that the structure of the introduction which was substantially rewritten in the previous revision reasonably overlaps with the structure that the Reviewer recommended. It is possible that each paragraph is longer than what the Reviewer had in mind, but we sincerely hope these can be appreciated as small differences in opinion.**

(2) Results

a. I find it hard to get an intuitive grasp of the exponential-logarithmic model. How does it react to different levels of surprise (absolute PEs) and noise

(sigma)? **It is worthwhile to highlight that models that we propose cannot track environmental volatility or noise which can only be done by Bayesian models. Essentially, the models will find the best trajectory of the learning rate versus prediction error relationship that fits the choices from the participants, considering that participant choice behaviour, and the magnitude of the prediction errors they would experience change with respect to different sources of uncertainty in the environment.**

What is the role of delta and lambda? **These parameters govern the trajectory of the weighting function. Both of these parameters control different aspects of the trajectory of the curve to find the best trajectory that explains the relationship between prediction errors and learning rates. Below, we are sharing 2 examples.**

As free parameters, how are they to be interpreted conceptually / at a cognitive level? In Fig.3, I can’t make out what the different values of delta and lambda are. Are they shown? If not, why not?

**The easiest way to conceptualise these parameters is again through the trajectory of the curve fitted to single participant data. It illustrates where the learning rates accelerate or decelerate above or below a linear relationship.**

**The sole purpose of the Figure 3 is to illustrate that it is possible to cover many different trajectories within the learning rate/prediction errors space, while also illustrating that this model can produce horizontal lines, i.e. constant learning rates if a constant learning rate assumption that is inherent in the Rescorla Wagner model is supported by the data. The nice bit of this model is that it can fully reduce to the Rescorla Wagner model if the choice behaviour supports a constant learning rate assumption. Figure 3 was generated by scanning through both of the parameter values and the evaluating the curve generated by the weighting function.**

**Below, we are providing an example of how each parameter influences the trajectory of the weighting curve when the other one is fixed to 1. Figure on the left, when Delta is fixed to one and lambda is varied from 0.005 to 3 (bluer tones indicate increasing parameter values). The figure on the right, when lambda is fixed to one and delta is varied from 0.005 to 3, again bluer lines indicate increasing parameter values. One could say, higher the lambda value the more sigmoidal the relationship will be as opposed to a constant learning rate, whereas higher the Delta, more parabolic the relationship between prediction errors and learning rates (although again we need to emphasise that these parameters work together)**

b. Details on the simulations are missing (number of simulations, parameters etc.) – as it is, I could not reproduce them. If full details take up too much space, this could go to the methods.

**We apologise for the omission. The simulations reported in Figure 4 was conducted in a single environment as the example task environment covers many possibilities of environmental volatility and noise and the trial sequence is reasonably lengthy. In the revised submission, we have added a single line to highlight that the simulation environment was generated once, whereas fitting of the models were done iteratively covering the whole parameter spaces, until the parameter combination which minimise the magnitude of the prediction error is found (akin to a maximum likelihood estimation, line 353). We will also make sure that the scripts related to the simulation are available in the online repository.**

c. As mentioned above, my main issue with this paper is that the authors do not show that their model is superior to existing models. But given theoretical assumptions, previous work, and the simplicity and elegance of their (exponential-logarithmic) model, it is actually surprising (to me) that it isn’t. I would encourage the authors to

**We thank the Reviewer for the globally positive feedback.**

i. try to find a simulated environment where their models do outperform the others, and/or

**Frankly speaking, my [corresponding author’s] initial reaction was that this exercise could lead to something like reverse-overfitting. Generally, considering that the differences between the models are somewhat negligibly small (our proposed models perform ever so slightly better or worse in different datasets), the fact that the non-linear models did not outperform the RW and PH by large margins did not seem problematic either. However, the Reviewer’s questions prompted curiosity and in this revision, we have now tested the monetary performance of the participants whose data is better explained by one model or the other. Additionally, we also generated more simulation environments randomly (1000 environments) and evaluated in what kind of environments are models would fit the data better, in line with the Reviewer’s recommendation. The updated results appear from line 557.**

ii. attempt an explanation as to why they do not in the existing synthetic and real datasets, and why some of the theoretical assumptions about how LR and PE should be related do not seem to hold. E.g., Fig. 8 seems to show very little adaptation of LR to PEs but rather strong interindividual variance. Likewise, in the same experiment, environmental noise did not influence LRs in stable environments. In addition, volatility did not influence LRs in the Behrens data. All of this is contrary to assumptions.

**We think the Reviewer raises some valid questions which are also at the forefront of reinforcement learning. For example, in a recent paper we have demonstrated that one of the reasons why human participants perform suboptimally (i.e. not in accordance to theoretic assumptions) in environments where volatility and noise are independently manipulated is because they make missattributions about the true source of uncertainty (i.e. mistaking noise for volatility), which can be understood/revealed by fitting of Bayesian models to participant choice behaviour (Pulcu & Browning, 2025: https://elifesciences.org/reviewed-preprints/103734). However we think these are separate questions than what we endeavoured to isolate in this paper, that is the non-linear relationship between learning rates and prediction errors, although we agree that the points raised by the Reviewer are related. With regards to trajectories shown in Figure 8, I think it nicely illustrates a mixture of parabolic and exponential curvatures and highlights the inter-individual variability as the Reviewer suggested. With regards to Behrens data, the effect of volatility disappears from all the models when the models are fitted continuously, which is also a fair assumption. We think the models identify an effect of volatility on learning rates when they are fitted separately to each block, which is the more common implementation in the field. We think this is a very valid and important conceptual question which requires further debate in the field, not something that we can resolve in this paper. However, rarely papers report findings from both approaches (i.e. continuous versus blockwise fitting, to be honest I have never seen a published example) and we think that our presentation of these findings back to back should stimulate discussion in the field about what is/should be the gold standard approach.**

d. I still find the model comparison procedure idiosyncratic and somewhat confusing. I would suggest that the authors make a reasoned choice for one single metric (that penalizes model complexity) and stick to it throughout the manuscript.

**In the current revision, we reported more AIC values across all experiments in an attempt to unify these reporting. We still kept some of the other model fitting metrics hoping that it can also inform the readers about the true model performance if we do not penalise for additional number of parameters. Globally AIC values suggest that our models fit ever so slightly better or worse depending on the dataset.**

e. I would like to see both model and parameter recovery for all models (though specifically for the two new models) (this may also help in answering 2.c).

**To reduce analysis fatigue, we decided not to perform further parameter recovery simulations for the cubic model, which did not perform well in Vaghi experiment, in our own data and in further simulations requested by the Reviewer. Obviously, poor model fitting from the onset would also impair parameter recoverability in a given environment so we thought this may be an inadvertantly misleading exercise. We had reported the parameter recovery of RW model in many of our previous papers (e.g. Pulcu et al., 2024) and the take-home message always is the same, as long as the inverse temperature term is not too low (i.e. choices being highly random) parameter recovery of these models are fairly robust in RL tasks and we thought this would not require further scrutiny.**

f. I encourage the authors to review their figure legends. It seems to me that they sometimes contain much more than descriptions and necessary explanations for the figure (e.g., Fig.2 line 235ff.). Perhaps they can move the rest to the main text, or if not relevant, omit it.

**We agree with the Reviewer that some of the figure legends may be long. However in computational neuroscience papers the most frustrating is trying to find out all the relevant information about a figure across the main manuscript, methods and Supplementary materials, quickly becomes a working memory capacity challenge. We think having lengthy legends for some figures will help making the figures self-contained and will also help readers from [neuro-]diverse backgrounds to follow the message that we try to convey easier. We hope that the Reviewer will sympathise with our opinion.**

Reviewer #3: Disclaimer:
This paper seems to be a new submission which was submitted before to the same journal (noted as a new manuscript ID). I was one of the original reviewers (Review #1) for ther early submission.
The authors did try to address as many review comments as possible. But in my view, this manuscript, in its current form, is still far from being ready to be published.
**In this revised submission, we made further changes to improve the manuscript based on all Reviewer feedback.**

At large, the writing style does not seem to be motivated as a scientific publication. In many places, the authors use phrases like “We think […]” “Let us start by …”, which, with due respect, are very much written as an essay rather than a academic report. If the authors were choosing to rewrite it, the manuscript will appear to be substantially different than the current one, which may again trigger the action as a new submission rather than a revision. I leave this for the editor to decide which is the best way to proceed.

**Number of our peers, including the other Reviewers in this journal, provided comments on this manuscript, and we never received any similar feedback. Below I am sharing examples from 2 papers using somewhat similar language (one from this journal)** <https://journals.plos.org/ploscompbiol/article?id=10.1371/journal.pcbi.1000254>

<https://www.pnas.org/doi/epdf/10.1073/pnas.1206569109>

Relatedly, there are multiple jumps in the flow of the writing. For example, the key concept of learning rate is mentioned before being properly explained/introduced. On line 83, it is the first time the term learning rate is mentioned, but only until line 93-94, learning rate is given a definition. Also, I would not use the acronym LR for learning rate, as, in the manuscript, there are both RL (reinforcement learning, which is a way more common acronym) and LR (learning rate), which is rather confusing to read.
**In the revised submission, the learning rates are now defined much earlier in the manuscript (line 104, also defined in the abstract prior to this point) and following the Reviewer’s suggestion we omitted using any acronyms for learning rates. We made further changes to improve the reading and flow of the manuscript, and just as before we consulted the opinion of our native-English colleagues at the University of Oxford with expertise in reinforcement learning.**

A rather critical issue is where the main model comes from. It seems as if one came up with a model out of no where, then it happened to work. Although there are indeed some assumptions (starting from line 176), there exists multiple non-linear functions that may also be plausible. Are there any theories, neuro/biological evidence to suggest the proposed model is the best choice?

**We appreciate a positive feedback from the Reviewer agreeing that our model works. As the Reviewer acknowledged, the model assumptions are outlined in the introduction running up to the Figure 1 where they are visually illustrated. As cited in the paper, these come from what we already know about the relationship between prediction errors and learning rates, and it is just that we happened to sit down to think about simple mathematical models that can account for these relationships. Some of these models (e.g. exponential logarithmic) come directly from behavioural economics. These were developed/proposed at a time when no neurobiological evidence existed (e.g. Kahneman & Tversky, Prospect Theory, Prelec Probability weighting). It is only logical to test the suitability of these models to the context of reinforcement learning due to similarities between inputs and outputs. I am personally surprised that no one looked into the suitability of these models previously. Through pupilometry analysis, we also provide physiological evidence in support of our proposal that change in learning rate speed is more likely to happen during the course of the trials rather than in a subsequent trial as the Pearce Hall model would suggest (pupil data is analysed by both models). As the Reviewer highlighted, there can be other plausible non-linear functions (here we have reported 2) and we hope that our paper will stimulate discussions in the field so that more models can become available to use in cognitive neuroscience.**

I also noticed that the authors have toned down that the proposed model is not superior to existing one, but rather a alternative. This somewhat undermines the value of this work, and impacts the potential to be eventually published in this journal.

**We do not agree with the Reviewer’s assessment. As we demonstrated across many tasks well-established models such as Rescorla Wagner and Pearce Hall do not always fit the data equally well. These differences, at least in our opinion, do not and cannot undermine the contribution of these models to the RL literature. Similarly, our models provide novel insights and a different way of thinking about learning rate problems, and solve a shortcoming of the most commonly implemented Rescorla Wagner model without [much] compromise.**

Moreover, some details are still lacking for the simulation. It seems that an algorithm (start from line 298) is used to minimize PE, without using functions such as softmax. But then how are actions/choices simulated? Some form of choice rule must have been applied? OR the model only cares about “value” rather than action?
**Here, the Reviewer is correct as simulating actions require many assumptions about choice rules and choice stochasticity which is not the main objective of the simulations, and we only focus on models’ ability to track expected values.**
Very last (sort of minor), the statement of “PEs are always between -1 and 1” (line 178 ) is incorrect. The range of PE is dependent on how outcome/reward is coded. If, as one example, in a task/model the reward ranges from 0 to 5, then the range of PE is -5 and 5.

**We completely agree with the Reviewer, and made a clarification statement to that bit of the manuscript.**
