# Supplementary material for: A nonlinear relationship between prediction errors and learning rates in human reinforcement-learning
Source: PLoS Comput Biol. 2025 Sep 12;21(9):e1013445. doi: 10.1371/journal.pcbi.1013445 (PMC12449023; doi:10.1371/journal.pcbi.1013445)
Supplement: Attachment — Submitted filename: PloS_CB_Reviewer_responses_R3.docx [file pcbi.1013445.s006.docx]

**Responses to the reviewers are typed in bold and carryovers from the article are in italic.**
Reviewer's Responses to Questions

**Comments to the Authors:**

Reviewer #2: I thank the authors for their clarifying comments. These have not, however, changed my fundamental view of the issues. As such, I am still unconvinced that the manuscript is ready for publication; however, given that the disagreements are well spelled out at this point, I do not believe a further round of reviews would necessarily be helpful and would leave it up to the editors to decide whether to go forward. Nonetheless, I provide a few more comments (including some reiterations of my previous comments) in the hope that the authors will find them useful.

Major
The motivation remains unclear to me. I’m not trying to be facetious, but I genuinely struggle to understand the paper's main aim – whether it aims to describe human learning accurately (which the authors suggest Bayesian models do better) or to provide more usable, intuitive models. If the latter, then conceptual/psychological interpretability of all free parameters is essential, as is superior model fit and model validation because they are necessary to be reasonably confident that the variable learning rates the models provide are meaningful. This is particularly important if the goal is to track individual differences in the relationship between learning rates and prediction errors – which their exponential-logarithmic model enables (which is great!) –, or to track learning rate signatures in the brain. At a minimum, the authors should demonstrate that the RW and exponential-logarithmic models are distinguishable in model recovery analyses using realistic parameter ranges (drawn e.g., from their simulations and reanalyses).

**We can confirm that the motivation behind our paper is the latter and consequently we highlighted this in the introduction section [from line 111]:**

*“The main motivation of our work is to inform a wider discussion in the field of RL towards development of more usable, relatable and intuitive computational models that can make investigations into the relationship between learning rates and prediction errors accessible to scientists across cognitive neuroscience, experimental psychology, and computational biology and psychiatry.”*

**In order to aid the parameter interpretability, we have revised our figure 3 in line with the Reviewer’s feedback, and we added 2 further panels to this figure so that the readers can fully understand how each parameter influences the learning rate trajectory.**

Minor
• There is no real justification for the non-traditional fitting approach (why not use generate-recover allowing traditional likelihood estimation?) and for minimizing prediction errors across the entire trajectory when this differs from experimental data analysis. As an experimentalist, this puzzles me. I would like to understand when this model would be preferable to other models, so simulations should examine simulated experimental data with plots showing both simulated and fitted behavior.

**As highlighted before, for the simulation, minimising prediction errors (i.e. Figure 4) is the most logical way from our perspective as we do not need to make any assumptions about the level of choice stochasticity which can easily interact with the performance of these models (even non linearly affecting certain models more so than others). It is evident that this is not a question that we are addressing in the current manuscript. The current approach actually aligns with the analysis of Vaghi data where participant choices were not discrete/binary. However, it is also not like we haven’t performed any generate-recover simulations (Supplementary Figure 9) in the traditional sense. The starting point of those simulations were the data from our own participants, therefore had the stochastic choice element inherently without needing to make arbitrary assumptions about it.**

**In the current revision, we further also expanded generate-recover simulations comparing Rescorla Wagner model and exponential logarithmic models as the Reviewer requested. We added a panel C to the Supplementary Figure 9 in which we generated choices by different models and fitted both the Rescorla Wagner model and the exponential logarithmic model. The result of this exercise suggested that:**

***“(C)*** *Results of generate recover simulations directly comparing the RW and exp-log models. Choices were generated by relying on the population mean of parameters as obtained from the main analysis, RW=[.5992 5.8259], exp-log=[.6723, .7045 & 5.8987]. After the generation of choices, the models were re-fitted and model probability evaluated. The confusion grid suggests that when choices were generated by the RW model, both models fit the data equally well, whereas when the choices were generated by the exp-log model, exp-log model fits the data better. Colour bar shows the likelihood of the model accounting for the synthetically generated choice data.”*

• It seems improper to cluster models by parameter count for model comparisons (lines 413ff). I would still urge the authors to consistently use model comparison that penalize complexity, including parameter number

**We understand that the small section referred to by the Reviewer becomes confusing and redundant in the light of null results from rmANOVA and further AIC results which penalise for model complexity reported further down in the same section, therefore we decided to remove the mentioned sentence altogether from the manuscript. We included model selection information with AICs (from line 429):**

*“Model comparison metrics which penalise for number of parameters such as Akaike’s Information Criterion favored the hybrid Pearce-Hall model (AIC=84.486) over Rescorla-Wagner model (AIC=84.888) and cubic model (AIC=84.931), and finally the exp-log model (AIC=85.726). Due to such small margins between the models, Bayes Omnibus risk calculated for these comparisons is 0.943 indicating that it is difficult to discriminate between model fits.”*

• I maintain that much information provided in the figure legends belongs in the main text (or it risks being overlooked)

**In this revised submission, we carried some text from each and every one of the main figure legends back to the body of the article, so that each of the figure legends is shorter than the previous submission. We kept the supplementary figure legends as before assuming that readers who would like to evaluate the supplementary figures would also read the corresponding legend immediately underneath.**

• Figure 3 could really use a legend identifying the delta and lambda combinations for at least 3-5 lines. I may be dense, but the psychological meanings of lambda and delta remain unclear to me (unlike the parabolic and cubic model parameters, which are well described).

**We have now revised this figure 3 in line with Reviewer’s suggestions, highlighting few horizontal trajectories and their specific parameter values. We added 2 further panels which were shared in our responses to the Reviewer in the previous review stage so that the readers can understand how each of these parameters contribute to the trajectory of the learning rate.**

• It would be helpful if Figure 6 could show trial-by-trial learning rates for both RW (flat) and exponential-logarithmic models to illustrate their differences, plus within-subject standard deviations of learning rates. Since the exponential-logarithmic model can reduce to RW under certain conditions, these comparisons would show how substantially they really differ.

**We could not fully follow the logic underlying the Reviewer’s suggestion. As the reviewer indicated, Rescorla Wagner model would produce a single learning rate, i.e. a flatline over the whole prediction error spectrum. Thinking that plotting how the learning rates fluctuate from the exponential logarithmic model (in comparison to other learning models which was already shown in Supplementary Figure 4 in a very similar task environment) from 25 participants will produce a lot of wiggly lines and clutter figure 6 which is reasonably clear and visually appealing in its current form with 3 panels, we decided to report the learning rate value from the Rescorla Wagner model along with its standard deviation and a corresponding interpretation about the differences between these models in the Figure 6 legend. Note that, these changes led to a figure legend which was shortened based on reviewer feedback becoming lengthier again, but perhaps still shorter than its length in the previous version.**

• Please have another sweep for typos, there were a few stray ones.

**We have shown every effort to make sure that there are no further typos in the manuscript, and would be happy to make further changes if there were still stray ones that we failed to detect (unless they originate from British versus American English, e.g. randomise vs randomize).**

• Finally, it would be really helpful if the authors highlighted the changes made in response to the reviewer comments in the response letter – this would save reviewers a lot of time.

**Thank you for the feedback, we have adopted that style in the current revision.**

Reviewer #3: Although I do not fully agree with the style of writing, which is in essence the authors' choice, and having paper published in such a style previously does not mean this is the preferred way to communicate science, I do not object this MS being published.

**We are happy to hear this positive feedback from the Reviewer.**
As the authors have claimed, they believe this model/approach can be very useful for the field.
However, the current open code on OSF is rather disorganized - neither a Readme file nor enough documentation was provided. I would strongly encourage the authors to tidy up the code and write clear documentations of their matlab scripts, before the manuscript can be eventually published. This way, I believe the field can benefit from it at large.

**Thank you very much for pointing these out, we have gone through the scripts to make sure that they are suitably commented, and we also provided a readme file for future users.**
